# Pladias platform: Technical description of the database structure

**DOI:** 10.3897/BDJ.10.e80167

**Published:** 2022-04-01

**Authors:** Petr Novotný, Josef Brůna, Milan Chytrý, Vojtěch Kalčík, Zdeněk Kaplan, Tomáš Kebert, Martin Rohn, Marcela Řezníčková, Milan Štech, Jan Wild

**Affiliations:** 1 Department of Biology Education, Faculty of Science, Charles University, Prague, Czech Republic Department of Biology Education, Faculty of Science, Charles University Prague Czech Republic; 2 Czech Academy of Sciences, Institute of Botany, Zámek 1, Průhonice, Czech Republic Czech Academy of Sciences, Institute of Botany, Zámek 1 Průhonice Czech Republic; 3 Department of Botany and Zoology, Faculty of Science, Masaryk University, Brno, Czech Republic Department of Botany and Zoology, Faculty of Science, Masaryk University Brno Czech Republic; 4 Department of Botany, Faculty of Science, University of South Bohemia, České Budějovice, Czech Republic Department of Botany, Faculty of Science, University of South Bohemia České Budějovice Czech Republic; 5 Babická 2379/1a, Praha, Czech Republic Babická 2379/1a Praha Czech Republic

**Keywords:** botany, Czech Republic, database, flora, occurrence, plant, relational database model, species, trait, tree hierarchy, vegetation

## Abstract

**Background:**

Digitising and aggregating local floristic data is a critical step in the study of biodiversity. The integrative web-based platform Pladias, designed to cover a wide range of data on vascular plants, was recently developed in the Czech Republic. The combination of occurrence data with species characteristics opens many opportunities for data analysis and synthesis.

**New information:**

This article describes the relational structure of the Pladias database service (PladiasDB) and the context of the platform architecture. The structure is relatively complex, as our goal was to cover: (i) species occurrence records, including their management, validation and export of revised species distribution maps, (ii) data on species characteristics with quality control tools using defined data types and (iii) separate user interfaces (UI) for professionals and the general public. We discuss the approaches chosen to model individual elements in PladiasDB and summarise the experience gained during the first five years of operation of the Pladias platform.

## Introduction

A new botanical database platform called Pladias has recently been established in the Czech Republic. It aggregates data on spontaneously occurring vascular plants, including species occurrence records from a dozen institutional data providers and hundreds of scientists and hobby botanists (Wild et al. 2019), data on plant characteristics and data on vegetation types, making it an effective tool for botanical syntheses in Central Europe (Chytrý et al. 2021). Pladias has been extensively used by the domestic botanical community during its five years of existence and became the main source of information about Czech flora. It continues to be filled by individual species occurrence records as well as batch imports from co-operating institutions. A clone of this database for bryophytes and lichens has been created and other clones are in preparation. Here, we describe the database structure to support further deployment. Wild et al. (2019) provides a more detailed introduction to the taxonomic approach, record validation mechanism and workflow for creating species distribution maps.

The Czech Republic has a long history of botanical research (Danihelka et al. 2017) and an active community of scientists, nature conservationists and hobby botanists. The plant diversity of the country is described in flora monographs (Hejný and Slavík 1988 et seq.), identification keys (Kubát et al. 2002, Kaplan et al. 2019), a plant checklist (Danihelka et al. 2012) and a vegetation monograph (Chytrý et al. 2007 et seq.). Against this background, we aimed to develop a national platform for botanical data management, based on local needs and context.

The creation of biological databases is a relatively common component of scientific projects, but it is often associated with subsequent problems. More than half of core biological databases face financial difficulties (Merali and Giles 2005). In addition to financial sustainability, other issues include staffing and community anchoring, i.e. whether the tool has established itself amongst users and whether it can continue to perform the required functions.

In designing the Pladias database, we considered the following key elements:

- the interoperability of species occurrence records, data on species characteristics and vegetation data;
- the ability to work dynamically with a hierarchical system of taxa to transfer values (e.g. if a species has only one subspecies in the Czech Republic, but data are collected for only one of these two hierarchical ranks);
- a unified, centrally managed taxon checklist allowing direct use of data for analysis, without requiring conversions between taxon concepts;
- optimisation for plant data at the expense of extensibility to other taxa;
- preservation of the history of changes in database records;
- consistent use of open-source technologies to ensure long-term financial sustainability.

Some of these design requirements differ from the principles accepted in the Biodiversity Information Standards data model (Access to Biological Collection Data Task Group 2007, TDWG; Kennedy et al. 2006) and articulated by Patterson et al. (2008). In particular, during the Pladias development, we intentionally did not adhere to the *Inclusive* principle (ability to cover all forms of life), *Concept-capable* principle (parallel taxon concepts that are simultaneously valid or not invalidated) and *Distributed organisation* principle, which concerns the implementation of data storage. The advantages and disadvantages of this approach are commented below. If data are shared in global databases aggregating biodiversity data in the future, it will be necessary to add an export module mapping internal structures to standardised formats of Darwin Core. However, we have not yet proceeded with this task due to the complexity of approaches to sharing plant occurrence data by different partners in the Czech Republic.

The aim of this article is to describe the internal structure of PladiasDB and to identify and discuss specific solutions to problems commonly encountered in biodiversity databases. We focus on the PladiasDB data schema, which is an expression of the general concept of the database and can serve as inspiration for further work.

## Project description

### Study area description

Database of botanical biodiversity at the national scale (Czech Republic).

### Design description

**Figure 1.**
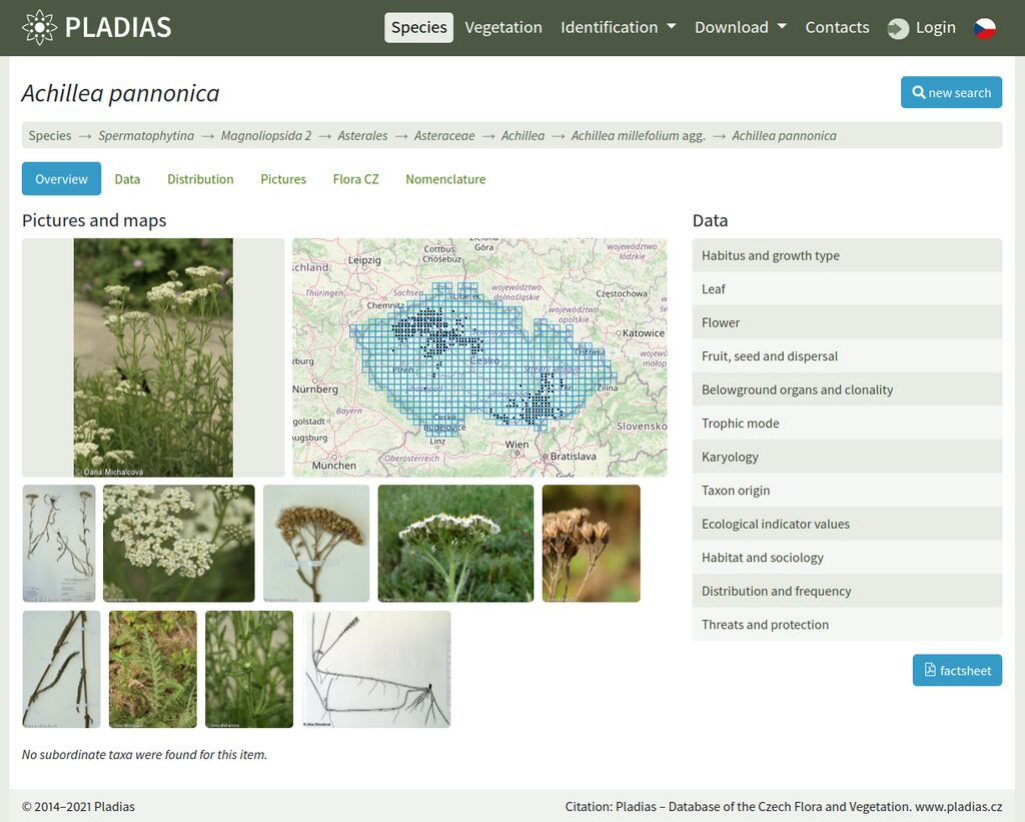
Screenshot of a taxon overview on the public portal pladias.cz.

**Figure 2.**
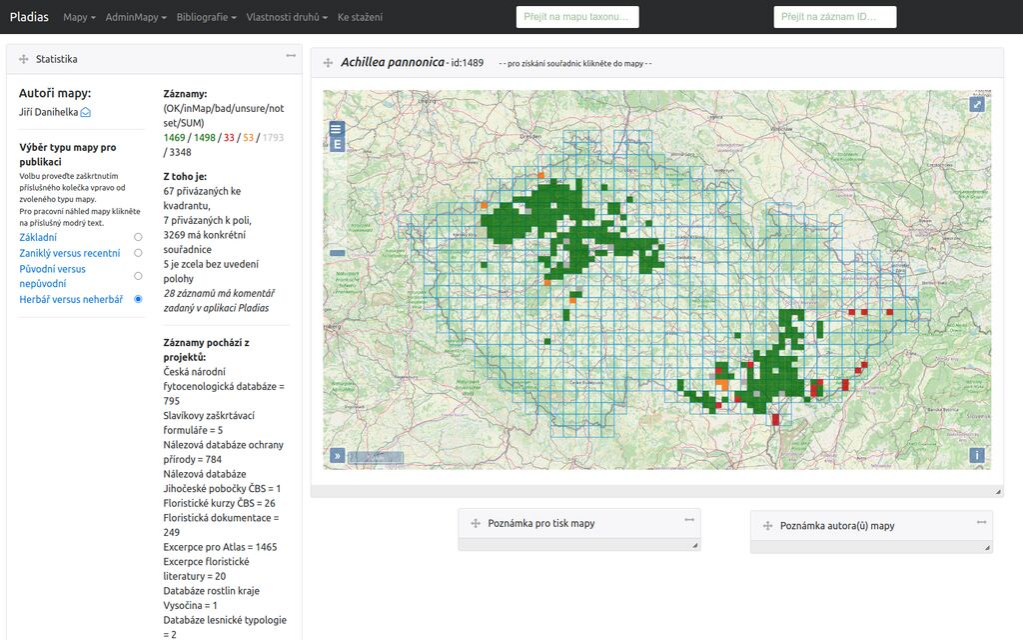
Screenshot of the online application with restricted access for researchers to work with the data at pladias.ibot.cas.cz.

**Figure 3.**
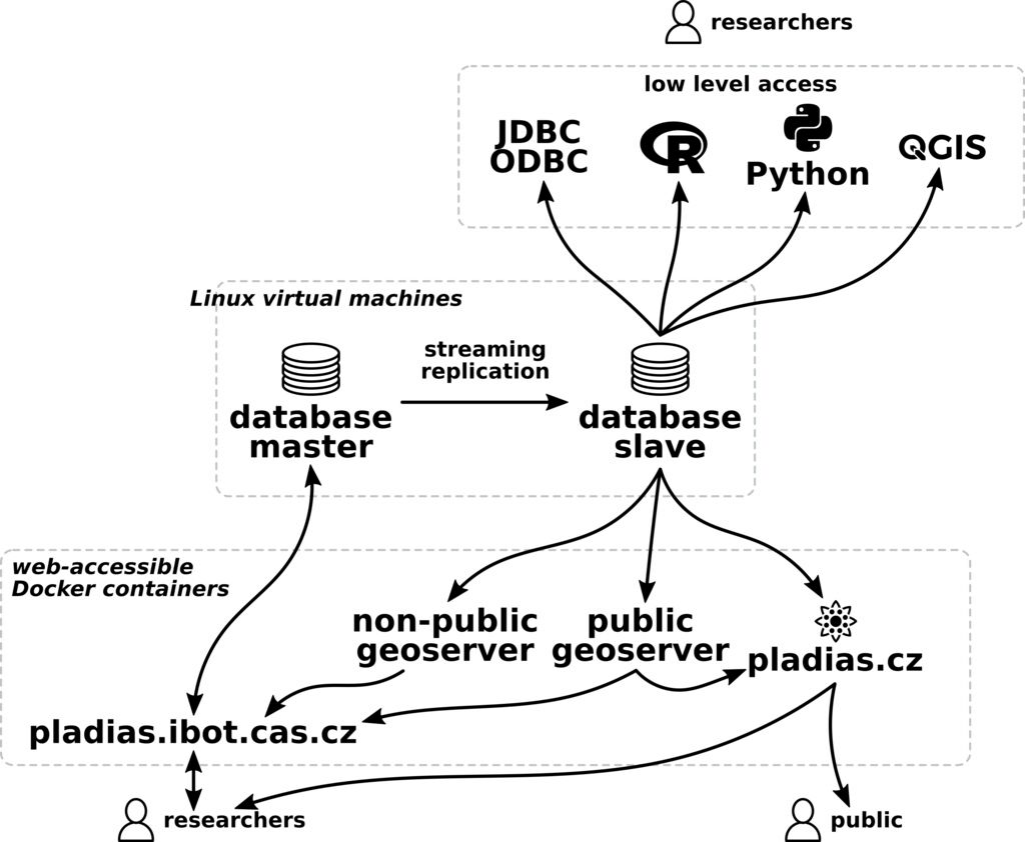
Pladias platform infrastructure. PladiasDB is stored on two servers with streaming replication to optimise server load. Data are edited through the pladias.ibot.cas.cz web application. Low-level read-only access is available to researchers via a direct connection to a secondary database server. Logos are the property of their respective owners. Sources: https://commons.wikimedia.org/wiki/File:QGIS_logo,_2017.svg, https://commons.wikimedia.org/wiki/File:R_logo.sv, https://commons.wikimedia.org/wiki/File:Python-logo-notext.svg.

The Pladias platform consists of several interconnected services designed as a web application. The public interface is based on the Nette framework (PHP) and is available at https://pladias.cz (Fig. 1). The interface for data management and editing by experts was created in the Play! framework (Java/Scala) and is available at https://pladias.ibot.cas.cz (Fig. 2). The data storage, PladiasDB, is a PostgreSQL relational database supplemented by the PostGIS extension, which allows it to work competently with spatial data. Spatial information is published using standard protocols, such as Web Map Service (WMS) or Web Feature Service (WFS). Two instances of Geoserver, one public and one private, are involved to facilitate access to restricted data on the distribution of rare species. An overview of the Pladias platform architecture is provided in Fig. 3. The public interface uses several front-end libraries, notably OpenLayers, Bootstrap and JQuery. Internal researchers also have access via a direct JDBC/ODBC database connection that can be used to access data from QGIS, R software and the like. All elements of the Pladias platform are based entirely on open-source solutions. The described stack of services, except for PostgreSQL, runs in containers using the Docker abstraction layer on the Linux-based production server hosted by the Institute of Botany of the Czech Academy of Sciences. There are two regular Linux-based database servers running in streaming replication to ensure performance and stability.

More than 80 tables of PladiasDB are divided into five table schemas: *atlas*, *geodata*, *measurements*, *pladias_functions* and *public*. The table schemas are based on content unity or as a separate functional unit within the platform. We use materialised views for computationally intensive operations, in particular the data for Geoserver are preprocessed and recomputed once a day.

The full SQL schema and the ER diagram showing the relationships amongst described tables are published online in the Git repository at https://git.sorbus.ibot.cas.cz/pladias-public/database-structure (Novotný 2022).

**Schema 1: atlas**

Schema *atlas* is a core for storing occurrence data. Data are imported as Excel files from specific sources (*atlas.projects*), resulting in the creation of a batch (*atlas.batch*). Any errors found by automatic checkpoints are reported to the user in the form of annotated Excel files (*atlas.excel*). Data that pass validation checkpoints are stored as species occurrence records (*atlas.records*). The import only allows records with a point location and is linked to the quadrant of the basic fields of the Mapping of the Flora of Central Europe (Ehrendorfer and Hamann 1965) quadrant. However, older records can be saved with location information that refers to the basic field (*atlas.records_squares*).

The remaining tables relate to record management and preparation of final versions of distribution maps for professional publications (e.g. Kaplan et al. 2015, Kaplan et al. 2021). All changes made since the dataset was imported are tracked in the database (*atlas.records_history*).

**Schema 2: geodata**

**Figure 4.**
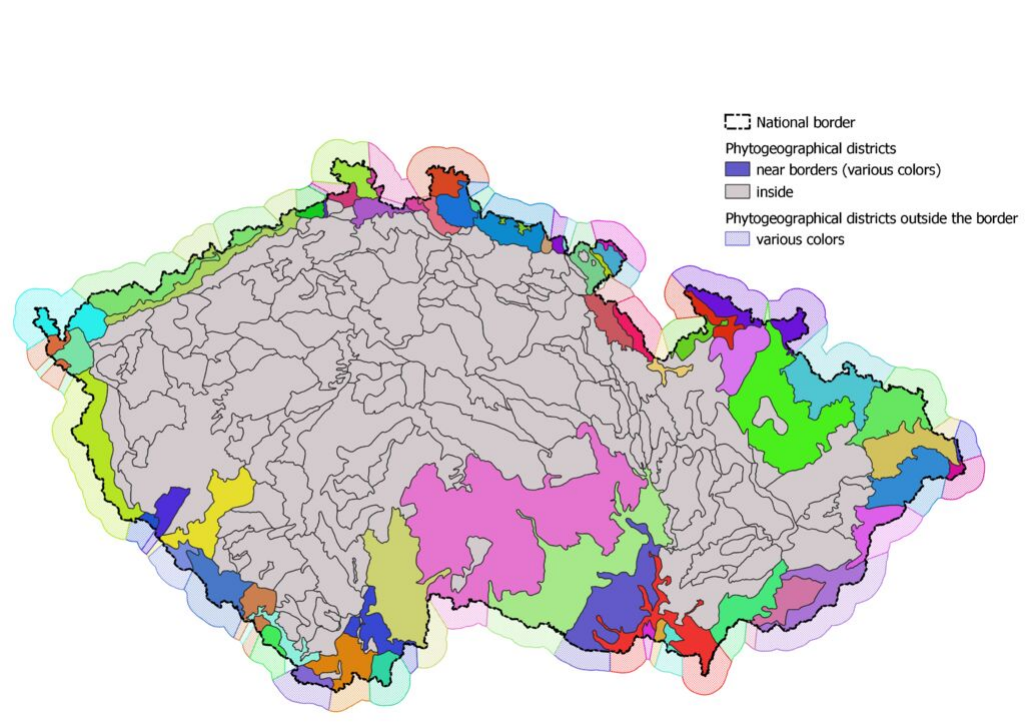
Phytogeographical districts of the Czech Republic and a buffer zone used for handling the records with coordinates falling slightly outside the national border. The districts at the national border are shown in colour.

Spatial data, except for the location of species occurrence records, are stored immutably in the *geodata* schema. The main code lists are the basic fieds ("squares") and quadrants (*geodata.squares_full*+*quadrants_full*) of the Mapping of the Flora of Central Europe, the phytogeographical division of the Czech Republic by Skalický (1988) (*geodata.phytochorions*) and the administrative division of the Czech Republic (*geodata.districts*). Pladias is a purely national database that only allows the import of species occurrence records from the territory of the Czech Republic with a buffer of 50 m beyond the national border, which should compensate for most localisation errors. For the validation of records with coordinates falling slightly outside the national border, especially when determining the phytogeographical districts, we use an approximation (*geodata.phytochorions_outside_cz*), based on a buffer around the national borders divided by perpendicular lines as shown in Fig. 4.

**Figure 5.**
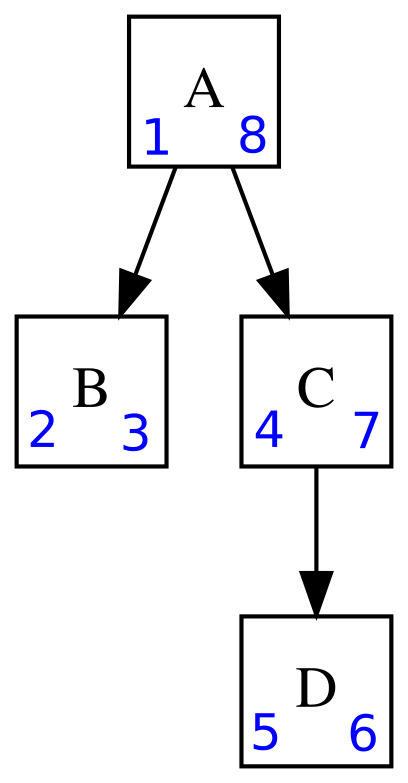
Nested set hierarchy model. *Left* and *right* attributes (in blue) are used to model the hierarchy of taxa A, B, C and D. These two attributes result from a pre-ordered (visiting the current node before traversing subtrees), depth-first (the tree walking is deepened as much as possible before going to the next sibling node) tree traversal. Such a traversal is topologically sorted, i.e. these two attributes store the tree structure and also the order of sibling nodes like order of species of the same genus. As a result of omitting recursive process, read queries are effective in this hierarchy model. For example, to obtain all the subordinate taxa of taxon A, we search for taxa that have *left* > 1 and *right* < 8 regardless of the size of the subtree. For reading efficiency, a redundant attribute *depth* can be added to describe the level of the hierarchy. Here, taxon A has a *depth* of 1 and taxon D has a *depth* of 3.

The administrative division of the country is a hierarchical list, similar to other lists in PladiasDB, for example, the lists of taxa and syntaxa (see Schema 5 *public*). Four approaches are generally used to store the tree structure in relational databases (Celko 2012), each of which has certain advantages and disadvantages. The specific choice of the tree modelling approach is influenced by the context of use. In the case of Pladias, we have consistently used the *Nested Set* modelling approach shown in Fig. 5. Its advantages, which we appreciated during the operation, include in particular:

- it features high reading speed and is very good at handling aggregation within subtrees, so that all operations in the hierarchy of core lists can be performed on the fly;
- it is able to easily query the hierarchy when mining data at the level of handwritten SQL queries;
- it is also understandable for non-technical users.

The third point is very important. Exports of tables, such as taxon checklists, include the key columns (*lft*, *rgt*, *depth*, e.g. *geodata.districts* table) describing the taxon hierarchy so that users can work independently with the hierarchy in a simple spreadsheet.

**Schema 3: measurements**

Besides the occurrence data, the second essential part of the database is the module containing plant characteristics (traits, environmental associations and other information). These data are stored in the *measurements* schema. Each characteristic is represented in the database by an envelope called feature (*measurements.features*) with metadata, such as measurement units or accepted values. The import is done by the user in the form of an Excel file and, after validation, forms a separate set of characteristics (*measurements.traits*), i.e. metadata of a particular import. The actual data on plant characteristics are stored in tables (*measurements.data_* *), depending on the data type (*measurements.datatypes*).

To fill in missing values, we use the taxonomic tree (see Chytrý et al. 2021 for details) in several ways (*measurements.inheritances*). Four levels of precision are then available in the tables of plant characteristics (*entry_type* column in the *measurements.data_* * tables):

- original = data imported by users
- inherited = data from hierarchically superior taxa (e.g. species data transferred to a subspecies); this is only used if the conditions defined by the data type, inheritance type and taxon hierarchy constellation are met
- aggregated = similar to inherited, but originating from hierarchically subordinate taxa
- composite = a combination of all the above points

Data reproducibility is important for practical scientific use of stored data, but in shared databases, data on plant characteristics are constantly being corrected and added. Several times we used the low-level approach of creating a snapshot of the entire database and storing it as a reference state for a particular analysis, but we have reached the limits of this approach. The snapshot of the database contains complete data, including users' contacts, their activities and other data that would need to be deleted before providing them for peer-reviews. Not only the stored data, but also partial changes in the database structure evolve, complicating automated processes in creating the snapshots described above. For data on plant characteristics, we therefore created the table *trait_export_snapshots*, which stores the complete data in a 2D structure with an annotation to which analysis the snapshot belongs. The snapshots are saved as an Excel file and made available to researchers via the web application.

**Schema 4: pladias_functions**

The *pladias_functions* schema provides a repository of PL/SQL functions implemented to work with data, mainly for administration, technical management and data mining. Although the functions always work with a particular table, the context of their calls is highly variable. A clear separation of the functions into isolated schemas facilitates the overview of the implemented functionality.

At the moment, all functions are dedicated to processing the *Nested Set* hierarchy in taxa and partially in syntaxa. The only table in this schema, *pladias_functions.mptt_taxons_errors* stores error messages for these functions.

**Schema 5: public**

The default PostgreSQL schema is used to store the core lists of the entire platform – data tables for user accounts, checklists of taxa (*public.taxons*) and syntaxa (*public.syntaxons*) including their associated data structures. Both checklists use the *Nested Set* structure to store the hierarchy, as described above in the *geodata* schema. The table names (*public.taxons*/*syntaxons*) intentionally use a grammatically incorrect form of the plural to follow the convention used for table names derived from the object in the application layer with the suffix "s". Therefore, we ignore irregular plural forms, such as *taxa* or *syntaxa*.

Taxon and syntaxon checklists are mandatory and no alternative concepts are allowed. This standardisation allows for efficient data management, which is particularly necessary because of the relational link to plant characteristics data at the database layer. To solve the issue of occasional changes in taxon names and delimitations, we adopted a synonym-derived solution. We arrange names representing different concepts in the table *public.synonyms*, which links names in the checklist with nomenclatural synonyms and names of taxa in different delimitations as adopted in important botanical monographs of the study area.

Some of the data stored in PladiasDB are original data, but most come from partially-aggregated sources (see Wild et al. 2019). These sources have very different licensing policies, which had to be accounted for by listing the licence for individual items, but also led us to create a security log of user activities. The *public.user_activity_log* table allows us to quantify each user's activities and capture potentially risky user behaviour.

## Web location (URIs)

Homepage: https://pladias.cz/en/

## Technical specification

Platform: PostgreSQL 12 + PostGIS 2.5

Programming language: SQL

Operational system: Linux

## Repository

Type: Git

Location: https://git.sorbus.ibot.cas.cz/pladias-public/database-structure

## Usage licence

### Usage licence

Other

### IP rights notes

The SQL structure of PladiasDB is licensed under

MIT Licence

Copyright (c) 2022 Pladias www.pladias.cz

Permission is hereby granted, free of charge, to any person obtaining a copy of this software and associated documentation files (the "Software"), to dealing with the Software without restriction, including without limitation the rights to use, copy, modify, merge, publish, distribute, sublicense and/or sell copies of the Software and to permit persons to whom the Software is furnished to do so, subject to the following conditions:

The above copyright notice and this permission notice shall be included in all copies or substantial portions of the Software.

THE SOFTWARE IS PROVIDED "AS IS", WITHOUT WARRANTY OF ANY KIND, EXPRESSED OR IMPLIED, INCLUDING BUT NOT LIMITED TO THE WARRANTIES OF MERCHANTABILITY, FITNESS FOR A PARTICULAR PURPOSE AND NON-INFRINGEMENT. IN NO EVENT SHALL THE AUTHORS OR COPYRIGHT HOLDERS BE LIABLE FOR ANY CLAIM, DAMAGES OR OTHER LIABILITY, WHETHER IN AN ACTION OF CONTRACT, TORT OR OTHERWISE, ARISING FROM, OUT OF OR IN CONNECTION WITH THE SOFTWARE OR THE USE OR OTHER DEALINGS IN THE SOFTWARE.

## Implementation

### Implements specification

**PladiasDB table description**

Tables that model basic objects are named with the plural form of the entity name. Tables modelling M:N (many-to-many) relationships use the naming convention "table1_table2". The SourceSQL code, the ER diagram and additional information are published in the Git repository.

**schema atlas** (Table 1)

**schema geodata** (Table 2)

**schema measurements** (Table 3)

**schema pladias_functions** (Table 4)

**schema public** (Table 5)

## Figures and Tables

**Table 1.** Database tables stored in the *atlas* schema. Counts of table rows as of 1 December 2021.

**Name**	**Description**	**Count of columns**	**Count of rows**
authors	persons who recorded plant occurrence in the field	4	14,283
batch	metadata of the occurrence data import batch	6	18,503
comments	users' comments on species occurrence records	11	25,647
csv_map_details	additional information used for map rendering in the map publishing workflow	6	941
excel	Excel files containing original species occurrence records (validated/imported)	9	17,452
herbariums	list of excerpted herbaria	13	267
institutions_users	administrator of a cooperating institution	2	0
pdf_map	PDF files containing distribution maps generated for use in printed publications	6	2,651
projects	set of batches/species occurrence records sharing the same source of funding or licensing conditions	6	15
projects_users	users allowed to import within a project	3	230
record_originality_status	list of states for species occurrence records' originality status	4	4
record_validation_status	list of states for species occurrence records' validity status	4	4
records	species occurrence records	40	13,635,402
records_authors	M:N link table	3	13,333,320
records_herbariums	M:N link table	2	506,159
records_history	log of any editing of species occurrence records after importing into the application	9	5,051,082
records_quadrants	M:N link table; Pladias allows old species occurrence records to be stored with inaccurate original locations by linking them to multiple adjacent quadrants. However, for ordinary users, the application layer only allows the import of point records, for which the linkage to the quadrant is calculated automatically	2	13,543,850
records_squares	M:N link table; see above table *public.records_quadrants*	2	38,604
taxon_mapsettings	settings and progress in the map publishing workflow	16	5,673
taxon_mapsettings_publication	list of available states of progress in the map publication	2	5
taxon_mapsettings_revision	list of available states of progress in the map revision	2	7
taxons_users	M:N link table; the assigned user is a map administrator of the taxon, including all of its hierarchically subordinated taxa, an has the right to determine validation status and change the taxon identification	3	1,413
users_comments	users being not asssigned as a map administrator can only comment on species occurrence records and propose changes for the revisers	2	4,925

**Table 2.** Database tables stored in the *geodata* schema. Counts of table rows as of 1 December 2021.

**Name**	**Description**	**Count of columns**	**Count of rows**
districts	administrative division of the Czech Republic	12	41,916
districts_depth	list of available states for administrative district hierarchy level	3	8
phytochorions	phytogeographical districts of the Czech Republic	7	215
phytochorions_outside_cz	approximation of phytogeographical division outside the country borders	4	89
quadrants_full	grid of mapping quadrants	10	40,000
regions	polygons of regions for specific projects	6	2
squares_full	grid of basic mapping fields ("squares")	4	10,000

**Table 3.** Database tables stored in the *measurements* schema. Counts of table rows as of 1 December 2021.

**Name**	**Description**	**Count of columns**	**Count of rows**
data_boolean	data on plant characteristics with Boolean data type	4	59,957
data_comment	pseudo-values for data on plant characteristics allowing comments on all data types	4	0
data_enum	data on plant characteristics with nominal or ordinal data types	8	10,296,633
data_enum_syntaxons	data on plant characteristics with specific data type that contains linking to the *public.syntaxons* table	7	37,688
data_integer	data on plant characteristics with numeric data types	6	196,510
data_interval_avg	extension of the previous numeric data type used for storing a broader set of values	9	165,804
data_month	data on plant characteristics with month data type	6	9,603
data_occurrence_frequency	data on species characteristics generated based on species occurrence records from the *atlas*module	6	27,792
data_percentage	data on plant characteristics with percentage data type	4	48,188
data_real	data on plant characteristics with decimal number values	4	53,514
data_real_multi	data on plant characteristics with decimal number values and multiplicit values per taxon	5	287,866
data_taxon_taxon_real	data on plant characteristics with data type storing numeric (real) value for a set of two taxa	5	129,476
data_unmeasurable	pseudo-values for data on plant characteristics allowing to mark values that are not measurable in the given context (for example, flower colour for ferns)	3	2,820
data_year	data on plant characteristics with year datatype	7	1,996
datatypes	list of implemented data types for data on plant characteristics	13	14
enumerates	metadata for nominal or ordinal lists of available values	6	104
enumerates_values	list of available values for nominal or ordinal data types	9	1,044
features	metadata for plant characteristics	25	291
inheritances	list of implemented inheritances, i.e. mechanisms for transferring values across a taxonomic tree	4	11
sections	hierarchical structure of features	10	38
trait_export_snapshots	storage for backups of data on plant characteristics used for reproducibility of analysis, flattened into a 2D structure and Excel file format	5	11
trait_visibility_status	list of available states for availability of data on plant characteristics in various export/publishing services of the Pladias platform	3	3
traits	metadata of specific series of data on plant characteristics	14	400
units	list of available units of measurement	6	16

**Table 4.** PL/pgSQL functions in the *pladias_functions* schema. See Git repository for input/output parameters.

**Name of the function**	**Description**
descendant_taxon()	provides the entire subtree of the taxon, including itself
get_parents_if_singleton()	recursive function that returns a continuous series of parent taxa that are monotypic
get_taxon_cloud()	aggregates descendant_taxon() and get_parents_if_singleton() function results; used for rendering maps of taxa aggregating records with different level of identification accuracy
mptt_syntaxons_appendchild()	add new syntaxon
mptt_taxons_appendchild()	add new taxon
mptt_taxons_delete_leaf()	delete taxon with no subordinate taxa
mptt_taxons_delete_subtree()	delete taxon and its subordinate taxa
mptt_taxons_get_depth()	numeric approach to reach *depth* of tree
mptt_taxons_get_error_code()	help for error messaging when using PL/pgSQL functions
mptt_taxons_move_subtree_before()	change the order of taxa belonging to a specific node. This function is used when changing the order of species listing inside one genus or other taxon; the parent (genus) remains the same, but the tree must be recalculated to change the order of species
mptt_taxons_move_subtree_real()	move a taxon subtree within a specific node; this function allows rebuilding the taxon tree by moving a taxon and all its subtaxa to a new parent (hierarchically superior taxon)
mptt_taxons_repair_depth()	recalculation of taxon nodes' *depth*

**Table 5.** Database tables stored in the *public* schema. Counts of table rows as of 1 December 2021.

**Name**	**Description**	**Count of columns**	**Count of rows**
downloads	static data provided in the web application	11	3
institutions	institutions providing the plant occurrence data	4	11
licenses	list of available licences for species occurrence records	3	6
publications	essential recent overview publications on the Czech flora	10	5
syntaxon_ranks	list of syntaxon hierarchy levels	5	5
syntaxons	core hierarchical list of syntaxa	33	674
taxon_ranks	list of taxon hierarchy levels	10	58
taxons	core hierarchical list of taxa	18	6,948
taxons_synonyms	taxon synonyms and invalidated taxon concept crosswalks mapped to the *public.publications*	7	18,485
user_activities	list of logged activities	2	21
user_activity_log	logging users' activity storage	7	4,170,445
user_settings	users' individual settings for web application	3	7,541
users	web application users	15	232
